# Diphen­yl[2-(2-pyridylamino­meth­yl)phen­yl]phosphine oxide

**DOI:** 10.1107/S1600536810014893

**Published:** 2010-04-28

**Authors:** Simón Hernández-Ortega, Fernando Cuenu Cabeza, Armando Cabrera-Ortiz

**Affiliations:** a Instituto de Química, Universidad Nacional Autónoma de México, Circuito Exterior, Ciudad Universitaria, México 04510, Mexico; b Laboratorio de Química Inorgánica y Catálisis, Programa de Química, Universidad del Quindío, Armenia, Quindío, Av. Bolivar calle 12 norte, Colombia

## Abstract

The title compound, C_24_H_21_N_2_OP, was obtained by reacting 2-amino­pyridine and 2-(diphenyl­phosphin­yl)benzaldehyde in ethanol. It crystallizes with two crystallographically independent mol­ecules in the asymmetric unit. The amino­pyridine units and the benzene ring bonded to the phosphine oxide P atom form dihedral angles of 88.58 (7) and 82.47 (9)° in the two mol­ecules. The crystal structure displays strong N—H⋯O and weak C—H⋯O hydrogen bonds along the *b* axis and C—H⋯π aromatic intra- and inter­molecular inter­actions.

## Related literature

For synthetic applications of pyridine-containing mol­ecules and amino­phosphines, see: Borah *et al.* (2010 ▶); Koprowski *et al.* (2002 ▶); Landaeta *et al.* (2006 ▶); Pfeiffer *et al.* (2000 ▶); Ribeiro *et al.* (2006 ▶). For similar structures, see: Pretorius *et al.* (2004 ▶); Sánchez *et al.* (2006 ▶).
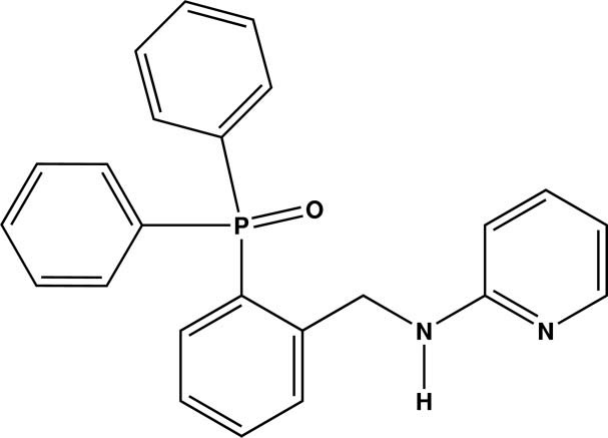


## Experimental

### 

#### Crystal data

C_24_H_21_N_2_OP
*M* *_r_* = 384.40
Monoclinic, 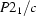

*a* = 24.130 (3) Å
*b* = 8.2397 (9) Å
*c* = 21.992 (2) Å
β = 109.108 (2)°
*V* = 4131.5 (8) Å^3^
*Z* = 8
Mo *K*α radiation
μ = 0.15 mm^−1^
*T* = 298 K
0.35 × 0.14 × 0.09 mm

#### Data collection

Bruker SMART APEX CCD area detector diffractometer
Absorption correction: multi-scan (*SADABS*; Bruker, 1999 ▶) *T*_min_ = 0.950, *T*_max_ = 0.987
32921 measured reflections
7544 independent reflections
4889 reflections with *I* > 2σ(*I*)
*R*_int_ = 0.062

#### Refinement

*R*[*F* ^2^ > 2σ(*F* ^2^)] = 0.056
*wR*(*F* ^2^) = 0.132
*S* = 1.06
7544 reflections
514 parameters
2 restraints
H atoms treated by a mixture of independent and constrained refinement
Δρ_max_ = 0.31 e Å^−3^
Δρ_min_ = −0.21 e Å^−3^

### 

Data collection: *SMART* (Bruker, 1999 ▶); cell refinement: *SAINT* (Bruker, 1999 ▶); data reduction: *SAINT*; program(s) used to solve structure: *SHELXTL* (Sheldrick, 2008 ▶); program(s) used to refine structure: *SHELXTL*; molecular graphics: *SHELXTL*; software used to prepare material for publication: *SHELXTL*.

## Supplementary Material

Crystal structure: contains datablocks I, global. DOI: 10.1107/S1600536810014893/bh2276sup1.cif

Structure factors: contains datablocks I. DOI: 10.1107/S1600536810014893/bh2276Isup2.hkl

Additional supplementary materials:  crystallographic information; 3D view; checkCIF report

## Figures and Tables

**Table 1:** Hydrogen-bond geometry (Å, °) *Cg*1, *Cg*2 and *Cg*3 are the centroids of the C13–C18, C39–C44 and N48,C47,C49–C52 rings, respectively.

*D*—H⋯*A*	*D*—H	H⋯*A*	*D*⋯*A*	*D*—H⋯*A*
N20—H20⋯O2^i^	0.90 (1)	1.98 (1)	2.872 (3)	177 (3)
N46—H46⋯O1^ii^	0.90 (1)	1.97 (1)	2.850 (3)	166 (3)
C4—H4⋯O1^iii^	0.93	2.53	3.329	144
C52—H52⋯O1^ii^	0.93	2.55	3.282	135
C3—H3⋯*Cg*1^iii^	0.93	2.95	3.627 (3)	131
C25—H25⋯*Cg*2	0.93	2.95	3.495 (5)	119
C30—H30⋯*Cg*3^iv^	0.93	2.93	3.772 (4)	152
C42—H42⋯*Cg*3^i^	0.93	2.96	3.746 (3)	154

Symmetry codes: (i) 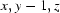; (ii) 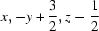; (iii) 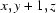; (iv) 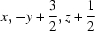.

**Fig. 1.**
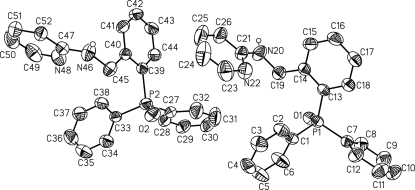
The two independent molecules in the title compound with the atom-labeling scheme. Displacement ellipsoids are shown at the 40% probability level.

**Fig. 2.**
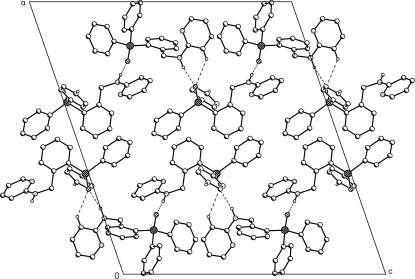
Crystal packing of the title compound. Hydrogen bonds are drawn as dashed lines.

## supplementary crystallographic information

### Comment

Nitrogen atoms of pyridine family heterocyclic compounds behave like excellent ligands in different reactions involving transition metal ions (Borah *et al.*, 2010). The presence of named ligands provides stability to the metal complex, even if the reaction is performed at high temperatures (Landaeta *et al.*, 2006; Ribeiro *et al.*, 2006). Aminophosphines type *P*,*N* are known to be hemilabile (Koprowski *et al.*, 2002; Pfeiffer *et al.*, 2000). In the quest for *P*,*N* ligands (Pretorius *et al.*, 2004; Sánchez *et al.*, 2006) that improve the stability of the metal complex and decrease the hemilability, we describe the crystal structure of the title compound.

The asymmetric unit contains two crystallographically independent molecules (*Z* = 8). The structure with numbering scheme is shown in Fig. 1. The molecules show different planar angles between the aromatic rings. The 2-aminopyridine moieties (N20···C26 in I and N46···C52 in II), are essentially planar and almost perpendicular to the benzene rings bonded to phosphino oxide (C13···C18 and C39···C44), the dihedral angles being 88.58 (7)° in I and 82.47 (9)° in II. These angles are similar to those of related structures found in the literature (*e.g.* 82.24°, Pretorius *et al.*, 2004).

Due the conformational geometry adopted by the molecules, in the crystal packing strong N—H···O intermolecular interactions and weak C—H···O as well as C—H···π intra and intermolecular interactions are present (see Tables and Fig. 2).

### Experimental

A solution of 2(diphenylphosphinyl)benzaldehyde (0.345 g, 1.189 mmol) in 5 ml of ethanol was added at room temperature to a solution of 2-aminopyridine (0.111 g, 1.189 mmol) in ethanol (5 ml). The resulting mixture was refluxed until the reaction was completed (5 hours). Then, solvent was evaporated, forming a light brown solid, which was filtered and washed with hexane. Yield: 0.408 g (89.3%).

### Refinement

Amine H atoms H20 and H46 were found in a difference map and refined with free coordinates and *U*_iso_(H) = 1.2*U*_eq_(N). N—H bond lengths were restrained to a sensible target value. Other H atoms were included in calculated positions (C—H = 0.93 or 0.97 Å), and refined using a riding model, with *U*_iso_(H) = 1.2*U*_eq_ of the carrier atom.

### Crystal data

**Table:**

C_24_H_21_N_2_OP	*F*(000) = 1616
*M**_r_* = 384.40	*D*_x_ = 1.236 Mg m^−^^3^
Monoclinic, *P*2_1_/*c*	Mo *K*α radiation, λ = 0.71073 Å
Hall symbol: -P 2ybc	Cell parameters from 6035 reflections
*a* = 24.130 (3) Å	θ = 2.2–23.8°
*b* = 8.2397 (9) Å	µ = 0.15 mm^−^^1^
*c* = 21.992 (2) Å	*T* = 298 K
β = 109.108 (2)°	Prism, colorless
*V* = 4131.5 (8) Å^3^	0.35 × 0.14 × 0.09 mm
*Z* = 8	

### Data collection

**Table:**

Bruker SMART APEX CCD area detector diffractometer	7544 independent reflections
Radiation source: fine-focus sealed tube	4889 reflections with *I* > 2σ(*I*)
graphite	*R*_int_ = 0.062
Detector resolution: 0.83 pixels mm^-1^	θ_max_ = 25.4°, θ_min_ = 1.8°
ω scans	*h* = −29→29
Absorption correction: multi-scan (*SADABS*; Bruker, 1999)	*k* = −9→9
*T*_min_ = 0.950, *T*_max_ = 0.987	*l* = −26→26
32921 measured reflections	

### Refinement

**Table:**

Refinement on *F*^2^	Primary atom site location: structure-invariant direct methods
Least-squares matrix: full	Secondary atom site location: difference Fourier map
*R*[*F*^2^ > 2σ(*F*^2^)] = 0.056	Hydrogen site location: inferred from neighbouring sites
*wR*(*F*^2^) = 0.132	H atoms treated by a mixture of independent and constrained refinement
*S* = 1.06	*w* = 1/[σ^2^(*F*_o_^2^) + (0.0576*P*)^2^] where *P* = (*F*_o_^2^ + 2*F*_c_^2^)/3
7544 reflections	(Δ/σ)_max_ < 0.001
514 parameters	Δρ_max_ = 0.31 e Å^−^^3^
2 restraints	Δρ_min_ = −0.21 e Å^−^^3^
0 constraints	

### Fractional atomic coordinates and isotropic or equivalent isotropic displacement parameters (Å^2^)

**Table:**

	*x*	*y*	*z*	*U*_iso_*/*U*_eq_	
P1	0.36928 (3)	0.34803 (8)	0.48820 (3)	0.04454 (19)	
O1	0.31222 (7)	0.26622 (19)	0.47912 (8)	0.0533 (5)	
C1	0.36101 (11)	0.5619 (3)	0.47160 (12)	0.0469 (6)	
C2	0.38693 (12)	0.6430 (3)	0.43397 (14)	0.0618 (8)	
H2	0.4128	0.5883	0.4178	0.074*	
C3	0.37526 (14)	0.8053 (3)	0.41956 (16)	0.0750 (9)	
H3	0.3931	0.8592	0.3938	0.090*	
C4	0.33743 (14)	0.8863 (3)	0.44333 (16)	0.0718 (9)	
H4	0.3294	0.9954	0.4336	0.086*	
C5	0.31182 (17)	0.8090 (4)	0.48059 (19)	0.0929 (12)	
H5	0.2860	0.8645	0.4965	0.112*	
C6	0.32358 (15)	0.6479 (4)	0.49535 (17)	0.0840 (10)	
H6	0.3060	0.5960	0.5218	0.101*	
C7	0.41605 (11)	0.3290 (3)	0.57041 (12)	0.0488 (6)	
C8	0.40174 (13)	0.2123 (3)	0.60849 (14)	0.0624 (8)	
H8	0.3692	0.1460	0.5909	0.075*	
C9	0.43526 (16)	0.1944 (4)	0.67180 (17)	0.0819 (10)	
H9	0.4251	0.1172	0.6971	0.098*	
C10	0.48385 (16)	0.2899 (5)	0.69802 (16)	0.0838 (10)	
H10	0.5069	0.2757	0.7408	0.101*	
C11	0.49844 (14)	0.4056 (4)	0.66157 (16)	0.0817 (10)	
H11	0.5314	0.4699	0.6796	0.098*	
C12	0.46437 (12)	0.4276 (4)	0.59788 (14)	0.0652 (8)	
H12	0.4739	0.5085	0.5735	0.078*	
C13	0.40760 (11)	0.2646 (3)	0.43713 (12)	0.0444 (6)	
C14	0.37875 (11)	0.2465 (3)	0.37016 (13)	0.0490 (6)	
C15	0.40969 (14)	0.1765 (3)	0.33377 (14)	0.0642 (8)	
H15	0.3915	0.1638	0.2897	0.077*	
C16	0.46664 (15)	0.1255 (3)	0.36129 (17)	0.0713 (9)	
H16	0.4863	0.0794	0.3356	0.086*	
C17	0.49465 (13)	0.1416 (3)	0.42583 (15)	0.0636 (8)	
H17	0.5332	0.1065	0.4442	0.076*	
C18	0.46515 (11)	0.2104 (3)	0.46333 (13)	0.0527 (7)	
H18	0.4842	0.2209	0.5074	0.063*	
C19	0.31515 (12)	0.2952 (4)	0.33873 (13)	0.0620 (8)	
H19A	0.2907	0.2172	0.3511	0.063 (8)*	
H19B	0.3092	0.3999	0.3558	0.060 (8)*	
N20	0.29567 (11)	0.3056 (3)	0.27000 (12)	0.0717 (7)	
H20	0.2706 (10)	0.229 (3)	0.2485 (13)	0.088 (11)*	
C21	0.30509 (12)	0.4386 (4)	0.23887 (14)	0.0626 (8)	
N22	0.34046 (11)	0.5533 (3)	0.27378 (12)	0.0762 (7)	
C23	0.34899 (16)	0.6856 (5)	0.2426 (2)	0.0977 (12)	
H23	0.3731	0.7665	0.2668	0.117*	
C24	0.3252 (2)	0.7099 (6)	0.1788 (2)	0.1167 (15)	
H24	0.3328	0.8039	0.1595	0.140*	
C25	0.28940 (18)	0.5911 (6)	0.14356 (19)	0.1085 (14)	
H25	0.2721	0.6039	0.0993	0.130*	
C26	0.27881 (14)	0.4540 (5)	0.17265 (16)	0.0819 (10)	
H26	0.2546	0.3727	0.1488	0.098*	
P2	0.16172 (3)	0.96647 (8)	0.17338 (3)	0.0487 (2)	
O2	0.21808 (8)	1.0515 (2)	0.20311 (8)	0.0655 (5)	
C27	0.14545 (12)	0.8307 (3)	0.22969 (12)	0.0517 (7)	
C28	0.09067 (13)	0.8167 (3)	0.23625 (13)	0.0598 (7)	
H28	0.0595	0.8773	0.2099	0.072*	
C29	0.08174 (15)	0.7131 (4)	0.28181 (14)	0.0728 (9)	
H29	0.0447	0.7053	0.2860	0.087*	
C30	0.1266 (2)	0.6228 (4)	0.32031 (16)	0.0897 (11)	
H30	0.1203	0.5525	0.3505	0.108*	
C31	0.1809 (2)	0.6356 (5)	0.31456 (18)	0.1041 (13)	
H31	0.2117	0.5737	0.3409	0.125*	
C32	0.19065 (15)	0.7397 (4)	0.27002 (16)	0.0855 (10)	
H32	0.2281	0.7487	0.2671	0.103*	
C33	0.10068 (11)	1.1037 (3)	0.14587 (12)	0.0479 (6)	
C34	0.10371 (13)	1.2529 (3)	0.17540 (14)	0.0632 (8)	
H34	0.1375	1.2822	0.2085	0.076*	
C35	0.05676 (16)	1.3588 (4)	0.15592 (18)	0.0799 (10)	
H35	0.0591	1.4591	0.1760	0.096*	
C36	0.00708 (16)	1.3166 (4)	0.10740 (18)	0.0848 (10)	
H36	−0.0244	1.3883	0.0947	0.102*	
C37	0.00316 (14)	1.1700 (4)	0.07738 (15)	0.0808 (10)	
H37	−0.0308	1.1418	0.0442	0.097*	
C38	0.04992 (13)	1.0642 (4)	0.09665 (14)	0.0659 (8)	
H38	0.0473	0.9644	0.0761	0.079*	
C39	0.15882 (10)	0.8468 (3)	0.10359 (12)	0.0458 (6)	
C40	0.17556 (10)	0.9141 (3)	0.05390 (12)	0.0472 (6)	
C41	0.17159 (12)	0.8183 (4)	0.00108 (13)	0.0600 (7)	
H41	0.1829	0.8612	−0.0321	0.072*	
C42	0.15121 (13)	0.6606 (4)	−0.00373 (14)	0.0674 (8)	
H42	0.1490	0.5988	−0.0398	0.081*	
C43	0.13435 (12)	0.5957 (3)	0.04431 (14)	0.0658 (8)	
H43	0.1204	0.4898	0.0412	0.079*	
C44	0.13816 (12)	0.6885 (3)	0.09777 (13)	0.0574 (7)	
H44	0.1266	0.6440	0.1306	0.069*	
C45	0.19811 (12)	1.0878 (3)	0.05873 (13)	0.0596 (7)	
H45A	0.1711	1.1572	0.0710	0.071*	
H45B	0.2356	1.0929	0.0930	0.071*	
N46	0.20546 (10)	1.1520 (3)	0.00087 (11)	0.0655 (7)	
H46	0.2420 (6)	1.166 (3)	−0.0005 (14)	0.079*	
C47	0.16033 (12)	1.2132 (3)	−0.04842 (13)	0.0517 (7)	
N48	0.10652 (10)	1.1985 (3)	−0.04558 (12)	0.0667 (7)	
C49	0.06305 (14)	1.2601 (4)	−0.0959 (2)	0.0875 (11)	
H49	0.0249	1.2524	−0.0950	0.105*	
C50	0.07112 (18)	1.3323 (4)	−0.1475 (2)	0.0947 (12)	
H50	0.0394	1.3712	−0.1811	0.114*	
C51	0.12719 (18)	1.3467 (4)	−0.14908 (15)	0.0808 (10)	
H51	0.1341	1.3965	−0.1839	0.097*	
C52	0.17227 (13)	1.2881 (3)	−0.09982 (13)	0.0600 (7)	
H52	0.2106	1.2974	−0.1000	0.072*	

### Atomic displacement parameters (Å^2^)

**Table:**

	*U*^11^	*U*^22^	*U*^33^	*U*^12^	*U*^13^	*U*^23^
P1	0.0453 (4)	0.0368 (4)	0.0537 (4)	−0.0034 (3)	0.0191 (3)	−0.0036 (3)
O1	0.0440 (10)	0.0464 (10)	0.0716 (12)	−0.0070 (8)	0.0217 (9)	−0.0086 (9)
C1	0.0500 (15)	0.0384 (14)	0.0544 (16)	−0.0029 (12)	0.0197 (13)	−0.0058 (12)
C2	0.0686 (19)	0.0444 (16)	0.083 (2)	−0.0057 (14)	0.0391 (16)	−0.0039 (15)
C3	0.099 (3)	0.0467 (18)	0.091 (2)	−0.0119 (17)	0.047 (2)	0.0037 (17)
C4	0.083 (2)	0.0346 (16)	0.094 (2)	−0.0013 (16)	0.0242 (19)	−0.0019 (16)
C5	0.119 (3)	0.0454 (19)	0.144 (3)	0.0115 (19)	0.083 (3)	0.000 (2)
C6	0.114 (3)	0.0493 (19)	0.120 (3)	0.0035 (18)	0.080 (2)	0.0017 (19)
C7	0.0506 (15)	0.0481 (15)	0.0527 (16)	−0.0001 (13)	0.0236 (13)	−0.0009 (13)
C8	0.0638 (18)	0.0644 (19)	0.064 (2)	−0.0008 (15)	0.0276 (16)	0.0057 (16)
C9	0.096 (3)	0.091 (3)	0.066 (2)	0.010 (2)	0.037 (2)	0.0181 (19)
C10	0.087 (3)	0.113 (3)	0.0507 (19)	0.017 (2)	0.0218 (19)	0.002 (2)
C11	0.074 (2)	0.099 (3)	0.062 (2)	−0.0126 (19)	0.0089 (18)	−0.016 (2)
C12	0.0689 (19)	0.0671 (19)	0.0585 (19)	−0.0118 (16)	0.0195 (16)	−0.0020 (15)
C13	0.0473 (15)	0.0331 (13)	0.0570 (17)	−0.0022 (11)	0.0228 (13)	−0.0001 (12)
C14	0.0566 (17)	0.0376 (14)	0.0546 (17)	−0.0023 (12)	0.0208 (14)	0.0009 (12)
C15	0.088 (2)	0.0537 (17)	0.0581 (18)	0.0001 (17)	0.0334 (17)	−0.0043 (15)
C16	0.090 (2)	0.0592 (19)	0.083 (2)	0.0169 (17)	0.053 (2)	0.0038 (17)
C17	0.0638 (19)	0.0553 (18)	0.081 (2)	0.0141 (15)	0.0363 (17)	0.0095 (17)
C18	0.0564 (17)	0.0451 (15)	0.0610 (17)	0.0031 (13)	0.0254 (14)	0.0045 (13)
C19	0.0643 (19)	0.0597 (19)	0.0576 (19)	−0.0071 (15)	0.0140 (15)	−0.0010 (15)
N20	0.0771 (18)	0.0701 (18)	0.0536 (16)	−0.0213 (15)	0.0020 (13)	−0.0056 (14)
C21	0.0540 (17)	0.071 (2)	0.0556 (19)	0.0042 (16)	0.0077 (15)	−0.0050 (17)
N22	0.0786 (18)	0.0664 (17)	0.0721 (17)	−0.0081 (14)	0.0091 (14)	0.0047 (15)
C23	0.092 (3)	0.077 (3)	0.116 (3)	−0.006 (2)	0.023 (2)	0.018 (2)
C24	0.099 (3)	0.127 (4)	0.117 (4)	0.009 (3)	0.025 (3)	0.057 (3)
C25	0.089 (3)	0.153 (4)	0.072 (3)	0.027 (3)	0.010 (2)	0.038 (3)
C26	0.067 (2)	0.106 (3)	0.059 (2)	0.012 (2)	0.0026 (17)	0.002 (2)
P2	0.0565 (4)	0.0461 (4)	0.0431 (4)	−0.0054 (3)	0.0156 (3)	−0.0045 (3)
O2	0.0632 (12)	0.0670 (12)	0.0586 (12)	−0.0165 (10)	0.0093 (9)	−0.0092 (10)
C27	0.0675 (18)	0.0471 (15)	0.0407 (15)	0.0036 (14)	0.0177 (14)	−0.0023 (12)
C28	0.073 (2)	0.0579 (18)	0.0476 (16)	−0.0048 (15)	0.0185 (14)	−0.0008 (14)
C29	0.090 (2)	0.079 (2)	0.0545 (19)	−0.0183 (19)	0.0302 (18)	−0.0005 (17)
C30	0.133 (3)	0.080 (2)	0.057 (2)	−0.007 (2)	0.032 (2)	0.0119 (18)
C31	0.123 (3)	0.102 (3)	0.085 (3)	0.034 (3)	0.031 (2)	0.044 (2)
C32	0.087 (2)	0.095 (3)	0.079 (2)	0.020 (2)	0.032 (2)	0.026 (2)
C33	0.0635 (17)	0.0411 (14)	0.0449 (15)	−0.0039 (13)	0.0254 (14)	−0.0032 (12)
C34	0.074 (2)	0.0526 (18)	0.0677 (19)	−0.0069 (16)	0.0290 (16)	−0.0058 (15)
C35	0.095 (3)	0.0499 (19)	0.106 (3)	0.0034 (19)	0.048 (2)	−0.0115 (19)
C36	0.086 (3)	0.077 (2)	0.095 (3)	0.027 (2)	0.035 (2)	0.007 (2)
C37	0.071 (2)	0.088 (2)	0.074 (2)	0.0184 (19)	0.0108 (17)	−0.011 (2)
C38	0.071 (2)	0.0598 (18)	0.0625 (19)	0.0109 (16)	0.0159 (16)	−0.0121 (15)
C39	0.0469 (15)	0.0439 (15)	0.0452 (15)	0.0003 (12)	0.0134 (12)	−0.0017 (12)
C40	0.0451 (14)	0.0540 (16)	0.0409 (14)	0.0046 (12)	0.0118 (12)	−0.0015 (13)
C41	0.0615 (18)	0.076 (2)	0.0440 (16)	0.0086 (16)	0.0189 (14)	0.0014 (15)
C42	0.078 (2)	0.070 (2)	0.0513 (18)	0.0105 (17)	0.0161 (16)	−0.0166 (16)
C43	0.077 (2)	0.0509 (17)	0.067 (2)	0.0012 (15)	0.0200 (16)	−0.0128 (16)
C44	0.0658 (18)	0.0517 (17)	0.0573 (18)	−0.0040 (14)	0.0236 (14)	−0.0043 (14)
C45	0.0651 (18)	0.0654 (18)	0.0494 (16)	−0.0061 (15)	0.0204 (14)	0.0058 (14)
N46	0.0513 (14)	0.0871 (18)	0.0620 (15)	0.0015 (14)	0.0239 (13)	0.0205 (14)
C47	0.0514 (17)	0.0470 (15)	0.0569 (17)	0.0022 (13)	0.0180 (14)	−0.0001 (13)
N48	0.0519 (15)	0.0629 (16)	0.0865 (19)	0.0028 (12)	0.0244 (14)	0.0023 (14)
C49	0.051 (2)	0.067 (2)	0.132 (3)	0.0039 (17)	0.012 (2)	−0.006 (2)
C50	0.087 (3)	0.075 (2)	0.094 (3)	0.020 (2)	−0.010 (2)	0.003 (2)
C51	0.109 (3)	0.069 (2)	0.059 (2)	0.019 (2)	0.020 (2)	0.0099 (17)
C52	0.0700 (19)	0.0587 (18)	0.0553 (18)	0.0100 (15)	0.0260 (16)	0.0065 (15)

### Geometric parameters (Å, °)

**Table:**

P1—O1	1.4866 (17)	P2—O2	1.4782 (18)
P1—C1	1.798 (2)	P2—C33	1.797 (3)
P1—C7	1.798 (3)	P2—C27	1.805 (3)
P1—C13	1.808 (2)	P2—C39	1.806 (3)
C1—C2	1.364 (3)	C27—C32	1.380 (4)
C1—C6	1.378 (4)	C27—C28	1.381 (4)
C2—C3	1.382 (4)	C28—C29	1.386 (4)
C2—H2	0.9300	C28—H28	0.9300
C3—C4	1.365 (4)	C29—C30	1.358 (4)
C3—H3	0.9300	C29—H29	0.9300
C4—C5	1.338 (4)	C30—C31	1.361 (5)
C4—H4	0.9300	C30—H30	0.9300
C5—C6	1.374 (4)	C31—C32	1.378 (5)
C5—H5	0.9300	C31—H31	0.9300
C6—H6	0.9300	C32—H32	0.9300
C7—C12	1.387 (4)	C33—C34	1.381 (3)
C7—C8	1.391 (3)	C33—C38	1.382 (4)
C8—C9	1.370 (4)	C34—C35	1.382 (4)
C8—H8	0.9300	C34—H34	0.9300
C9—C10	1.372 (5)	C35—C36	1.363 (4)
C9—H9	0.9300	C35—H35	0.9300
C10—C11	1.364 (4)	C36—C37	1.365 (4)
C10—H10	0.9300	C36—H36	0.9300
C11—C12	1.385 (4)	C37—C38	1.379 (4)
C11—H11	0.9300	C37—H37	0.9300
C12—H12	0.9300	C38—H38	0.9300
C13—C18	1.391 (3)	C39—C44	1.387 (3)
C13—C14	1.416 (3)	C39—C40	1.398 (3)
C14—C15	1.386 (3)	C40—C41	1.382 (3)
C14—C19	1.516 (4)	C40—C45	1.522 (3)
C15—C16	1.374 (4)	C41—C42	1.381 (4)
C15—H15	0.9300	C41—H41	0.9300
C16—C17	1.364 (4)	C42—C43	1.360 (4)
C16—H16	0.9300	C42—H42	0.9300
C17—C18	1.376 (3)	C43—C44	1.380 (4)
C17—H17	0.9300	C43—H43	0.9300
C18—H18	0.9300	C44—H44	0.9300
C19—N20	1.431 (3)	C45—N46	1.442 (3)
C19—H19A	0.9700	C45—H45A	0.9700
C19—H19B	0.9700	C45—H45B	0.9700
N20—C21	1.350 (4)	N46—C47	1.357 (3)
N20—H20	0.895 (10)	N46—H46	0.899 (10)
C21—N22	1.335 (3)	C47—N48	1.326 (3)
C21—C26	1.391 (4)	C47—C52	1.398 (4)
N22—C23	1.339 (4)	N48—C49	1.350 (4)
C23—C24	1.346 (5)	C49—C50	1.350 (5)
C23—H23	0.9300	C49—H49	0.9300
C24—C25	1.366 (6)	C50—C51	1.370 (5)
C24—H24	0.9300	C50—H50	0.9300
C25—C26	1.363 (5)	C51—C52	1.348 (4)
C25—H25	0.9300	C51—H51	0.9300
C26—H26	0.9300	C52—H52	0.9300
			
O1—P1—C1	112.39 (11)	O2—P2—C33	112.62 (12)
O1—P1—C7	110.16 (11)	O2—P2—C27	111.44 (12)
C1—P1—C7	106.34 (12)	C33—P2—C27	106.42 (12)
O1—P1—C13	112.55 (10)	O2—P2—C39	114.12 (11)
C1—P1—C13	106.98 (11)	C33—P2—C39	104.94 (11)
C7—P1—C13	108.14 (12)	C27—P2—C39	106.73 (12)
C2—C1—C6	117.7 (2)	C32—C27—C28	118.0 (3)
C2—C1—P1	124.1 (2)	C32—C27—P2	118.3 (2)
C6—C1—P1	118.0 (2)	C28—C27—P2	123.6 (2)
C1—C2—C3	120.9 (3)	C27—C28—C29	120.5 (3)
C1—C2—H2	119.5	C27—C28—H28	119.7
C3—C2—H2	119.5	C29—C28—H28	119.7
C4—C3—C2	119.8 (3)	C30—C29—C28	120.5 (3)
C4—C3—H3	120.1	C30—C29—H29	119.8
C2—C3—H3	120.1	C28—C29—H29	119.8
C5—C4—C3	120.2 (3)	C29—C30—C31	119.7 (3)
C5—C4—H4	119.9	C29—C30—H30	120.2
C3—C4—H4	119.9	C31—C30—H30	120.2
C4—C5—C6	120.2 (3)	C30—C31—C32	120.5 (3)
C4—C5—H5	119.9	C30—C31—H31	119.8
C6—C5—H5	119.9	C32—C31—H31	119.8
C5—C6—C1	121.2 (3)	C31—C32—C27	120.8 (3)
C5—C6—H6	119.4	C31—C32—H32	119.6
C1—C6—H6	119.4	C27—C32—H32	119.6
C12—C7—C8	118.8 (3)	C34—C33—C38	118.4 (3)
C12—C7—P1	123.3 (2)	C34—C33—P2	119.4 (2)
C8—C7—P1	117.9 (2)	C38—C33—P2	122.3 (2)
C9—C8—C7	120.4 (3)	C33—C34—C35	120.3 (3)
C9—C8—H8	119.8	C33—C34—H34	119.9
C7—C8—H8	119.8	C35—C34—H34	119.9
C8—C9—C10	120.3 (3)	C36—C35—C34	120.2 (3)
C8—C9—H9	119.9	C36—C35—H35	119.9
C10—C9—H9	119.9	C34—C35—H35	119.9
C11—C10—C9	120.2 (3)	C35—C36—C37	120.5 (3)
C11—C10—H10	119.9	C35—C36—H36	119.7
C9—C10—H10	119.9	C37—C36—H36	119.7
C10—C11—C12	120.3 (3)	C36—C37—C38	119.4 (3)
C10—C11—H11	119.9	C36—C37—H37	120.3
C12—C11—H11	119.9	C38—C37—H37	120.3
C11—C12—C7	120.0 (3)	C37—C38—C33	121.2 (3)
C11—C12—H12	120.0	C37—C38—H38	119.4
C7—C12—H12	120.0	C33—C38—H38	119.4
C18—C13—C14	118.8 (2)	C44—C39—C40	119.2 (2)
C18—C13—P1	120.6 (2)	C44—C39—P2	120.30 (19)
C14—C13—P1	120.59 (19)	C40—C39—P2	120.45 (19)
C15—C14—C13	118.0 (2)	C41—C40—C39	118.2 (2)
C15—C14—C19	120.2 (2)	C41—C40—C45	121.4 (2)
C13—C14—C19	121.7 (2)	C39—C40—C45	120.4 (2)
C16—C15—C14	121.5 (3)	C42—C41—C40	121.8 (3)
C16—C15—H15	119.2	C42—C41—H41	119.1
C14—C15—H15	119.2	C40—C41—H41	119.1
C17—C16—C15	120.8 (3)	C43—C42—C41	120.0 (3)
C17—C16—H16	119.6	C43—C42—H42	120.0
C15—C16—H16	119.6	C41—C42—H42	120.0
C16—C17—C18	119.1 (3)	C42—C43—C44	119.4 (3)
C16—C17—H17	120.4	C42—C43—H43	120.3
C18—C17—H17	120.4	C44—C43—H43	120.3
C17—C18—C13	121.7 (3)	C43—C44—C39	121.4 (3)
C17—C18—H18	119.1	C43—C44—H44	119.3
C13—C18—H18	119.1	C39—C44—H44	119.3
N20—C19—C14	115.5 (2)	N46—C45—C40	115.4 (2)
N20—C19—H19A	108.4	N46—C45—H45A	108.4
C14—C19—H19A	108.4	C40—C45—H45A	108.4
N20—C19—H19B	108.4	N46—C45—H45B	108.4
C14—C19—H19B	108.4	C40—C45—H45B	108.4
H19A—C19—H19B	107.5	H45A—C45—H45B	107.5
C21—N20—C19	122.2 (3)	C47—N46—C45	122.9 (2)
C21—N20—H20	120 (2)	C47—N46—H46	117.7 (19)
C19—N20—H20	117 (2)	C45—N46—H46	118.8 (19)
N22—C21—N20	117.7 (3)	N48—C47—N46	118.1 (3)
N22—C21—C26	121.6 (3)	N48—C47—C52	122.9 (3)
N20—C21—C26	120.7 (3)	N46—C47—C52	119.1 (2)
C21—N22—C23	117.3 (3)	C47—N48—C49	115.9 (3)
N22—C23—C24	124.8 (4)	C50—C49—N48	124.6 (3)
N22—C23—H23	117.6	C50—C49—H49	117.7
C24—C23—H23	117.6	N48—C49—H49	117.7
C23—C24—C25	117.4 (4)	C49—C50—C51	118.4 (3)
C23—C24—H24	121.3	C49—C50—H50	120.8
C25—C24—H24	121.3	C51—C50—H50	120.8
C26—C25—C24	120.6 (4)	C52—C51—C50	119.5 (3)
C26—C25—H25	119.7	C52—C51—H51	120.3
C24—C25—H25	119.7	C50—C51—H51	120.3
C25—C26—C21	118.4 (4)	C51—C52—C47	118.8 (3)
C25—C26—H26	120.8	C51—C52—H52	120.6
C21—C26—H26	120.8	C47—C52—H52	120.6
			
O1—P1—C1—C2	−132.3 (2)	O2—P2—C27—C32	−40.9 (3)
C7—P1—C1—C2	107.1 (2)	C33—P2—C27—C32	−164.1 (2)
C13—P1—C1—C2	−8.3 (3)	C39—P2—C27—C32	84.3 (2)
O1—P1—C1—C6	43.9 (3)	O2—P2—C27—C28	136.7 (2)
C7—P1—C1—C6	−76.7 (3)	C33—P2—C27—C28	13.6 (3)
C13—P1—C1—C6	167.9 (2)	C39—P2—C27—C28	−98.1 (2)
C6—C1—C2—C3	−0.9 (4)	C32—C27—C28—C29	−0.5 (4)
P1—C1—C2—C3	175.3 (2)	P2—C27—C28—C29	−178.2 (2)
C1—C2—C3—C4	0.2 (5)	C27—C28—C29—C30	−0.5 (4)
C2—C3—C4—C5	0.2 (5)	C28—C29—C30—C31	0.7 (5)
C3—C4—C5—C6	0.2 (6)	C29—C30—C31—C32	0.1 (6)
C4—C5—C6—C1	−1.0 (6)	C30—C31—C32—C27	−1.1 (6)
C2—C1—C6—C5	1.3 (5)	C28—C27—C32—C31	1.3 (5)
P1—C1—C6—C5	−175.2 (3)	P2—C27—C32—C31	179.1 (3)
O1—P1—C7—C12	−161.6 (2)	O2—P2—C33—C34	−26.9 (2)
C1—P1—C7—C12	−39.6 (3)	C27—P2—C33—C34	95.5 (2)
C13—P1—C7—C12	75.0 (2)	C39—P2—C33—C34	−151.6 (2)
O1—P1—C7—C8	16.5 (2)	O2—P2—C33—C38	154.4 (2)
C1—P1—C7—C8	138.5 (2)	C27—P2—C33—C38	−83.2 (2)
C13—P1—C7—C8	−106.9 (2)	C39—P2—C33—C38	29.7 (2)
C12—C7—C8—C9	−0.6 (4)	C38—C33—C34—C35	0.2 (4)
P1—C7—C8—C9	−178.8 (2)	P2—C33—C34—C35	−178.6 (2)
C7—C8—C9—C10	−1.0 (5)	C33—C34—C35—C36	0.1 (5)
C8—C9—C10—C11	1.3 (5)	C34—C35—C36—C37	−0.3 (5)
C9—C10—C11—C12	0.0 (5)	C35—C36—C37—C38	0.2 (5)
C10—C11—C12—C7	−1.7 (5)	C36—C37—C38—C33	0.1 (5)
C8—C7—C12—C11	2.0 (4)	C34—C33—C38—C37	−0.3 (4)
P1—C7—C12—C11	−179.9 (2)	P2—C33—C38—C37	178.5 (2)
O1—P1—C13—C18	−126.2 (2)	O2—P2—C39—C44	132.3 (2)
C1—P1—C13—C18	109.8 (2)	C33—P2—C39—C44	−103.9 (2)
C7—P1—C13—C18	−4.3 (2)	C27—P2—C39—C44	8.8 (2)
O1—P1—C13—C14	50.9 (2)	O2—P2—C39—C40	−49.7 (2)
C1—P1—C13—C14	−73.0 (2)	C33—P2—C39—C40	74.1 (2)
C7—P1—C13—C14	172.84 (19)	C27—P2—C39—C40	−173.3 (2)
C18—C13—C14—C15	−0.5 (3)	C44—C39—C40—C41	−1.0 (4)
P1—C13—C14—C15	−177.76 (18)	P2—C39—C40—C41	−178.93 (19)
C18—C13—C14—C19	177.0 (2)	C44—C39—C40—C45	179.7 (2)
P1—C13—C14—C19	−0.2 (3)	P2—C39—C40—C45	1.7 (3)
C13—C14—C15—C16	0.1 (4)	C39—C40—C41—C42	0.6 (4)
C19—C14—C15—C16	−177.5 (3)	C45—C40—C41—C42	180.0 (3)
C14—C15—C16—C17	0.3 (4)	C40—C41—C42—C43	0.0 (4)
C15—C16—C17—C18	−0.2 (4)	C41—C42—C43—C44	−0.4 (4)
C16—C17—C18—C13	−0.3 (4)	C42—C43—C44—C39	0.0 (4)
C14—C13—C18—C17	0.6 (4)	C40—C39—C44—C43	0.7 (4)
P1—C13—C18—C17	177.9 (2)	P2—C39—C44—C43	178.6 (2)
C15—C14—C19—N20	−16.3 (4)	C41—C40—C45—N46	9.8 (4)
C13—C14—C19—N20	166.2 (2)	C39—C40—C45—N46	−170.9 (2)
C14—C19—N20—C21	−82.7 (4)	C40—C45—N46—C47	81.3 (3)
C19—N20—C21—N22	9.1 (4)	C45—N46—C47—N48	−6.7 (4)
C19—N20—C21—C26	−171.1 (3)	C45—N46—C47—C52	173.5 (2)
N20—C21—N22—C23	−179.2 (3)	N46—C47—N48—C49	−179.5 (3)
C26—C21—N22—C23	1.0 (4)	C52—C47—N48—C49	0.3 (4)
C21—N22—C23—C24	−0.9 (5)	C47—N48—C49—C50	0.6 (5)
N22—C23—C24—C25	0.5 (7)	N48—C49—C50—C51	−0.9 (6)
C23—C24—C25—C26	−0.1 (6)	C49—C50—C51—C52	0.4 (5)
C24—C25—C26—C21	0.2 (6)	C50—C51—C52—C47	0.3 (5)
N22—C21—C26—C25	−0.7 (5)	N48—C47—C52—C51	−0.7 (4)
N20—C21—C26—C25	179.5 (3)	N46—C47—C52—C51	179.1 (3)

### Hydrogen-bond geometry (Å, °)

**Table:**

Cg1, Cg2 and Cg3 are the centroids of the C13–C18, C39–C44 and N48,C47,C49–C52 rings, respectively.

**Table:**

*D*—H···*A*	*D*—H	H···*A*	*D*···*A*	*D*—H···*A*
N20—H20···O2^i^	0.90 (1)	1.98 (1)	2.872 (3)	177 (3)
N46—H46···O1^ii^	0.90 (1)	1.97 (1)	2.850 (3)	166 (3)
C4—H4···O1^iii^	0.93	2.53	3.329	144
C52—H52···O1^ii^	0.93	2.55	3.282	135
C3—H3···Cg1^iii^	0.93	2.95	3.627 (3)	131
C25—H25···Cg2	0.93	2.95	3.495 (5)	119
C30—H30···Cg3^iv^	0.93	2.93	3.772 (4)	152
C42—H42···Cg3^i^	0.93	2.96	3.746 (3)	154

Symmetry codes: (i) *x*, *y*−1, *z*; (ii) *x*, −*y*+3/2, *z*−1/2; (iii) *x*, *y*+1, *z*; (iv) *x*, −*y*+3/2, *z*+1/2.
